# Biological sex influences bilateral transcortical reflexes during unilateral upper extremity exercise in healthy adults

**DOI:** 10.14814/phy2.70860

**Published:** 2026-04-06

**Authors:** Olga Dubey, Michael A. Petrie, Richard K. Shields

**Affiliations:** ^1^ Department of Physical Therapy and Rehabilitation Science Carver College of Medicine, University of Iowa Iowa City Iowa USA

**Keywords:** cross‐transcortical communication, long‐latency response, neuromuscular control, sex differences

## Abstract

The transcortical long‐latency reflex (LLR) is a rapid feedback response that is thought to help stabilize movement following sudden disturbances and is a critical mechanism enabling precise upper limb control during unexpected perturbations. It occurs 50–150 ms after limb displacement and integrates both spinal and supraspinal circuits. Despite extensive research on reflexes during movement, sex‐specific differences in LLR responses remain underexplored. Importantly, while bilateral reflex responses have been documented when both arms contribute to a shared movement goal, cross‐transcortical communication in relaxed, nonparticipating limbs during unilateral perturbations has not been investigated. This study examined sex differences in visuomotor tracking accuracy and transcortical reflex responses during exercise induced perturbations in 40 healthy participants (20 males and 20 females). Participants performed elbow flexion/extension tracking movements using their nondominant arm while the contralateral arm remained at rest. Unexpected perturbations were delivered by removing brake resistance during early elbow flexion, with surface electromyography recording muscle activity from bilateral triceps and biceps muscles. Results revealed distinct sex‐specific neuromuscular control strategies: females employed mainly feed forward co‐activation strategies, while males utilized reactive fast feedback LLR control strategies. Novel cross‐transcortical responses were detected in the nonparticipating contralateral arm, with sex‐specific bilateral motor control patterns emerging during unilateral perturbations. These findings advance our understanding of upper extremity movement control across sex during upper limb training and have implications for developing personalized skill training exercise programs for males and females.

## Graphical abstract

Strategies to Respond to a Perturbation are Distinct Among Females and Males.

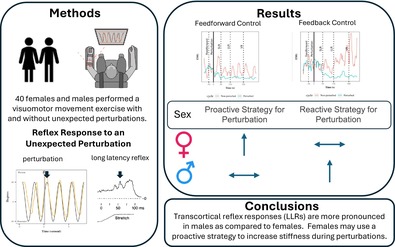

## 1 INTRODUCTION

Humans exhibit remarkable precision when exercising their upper limbs, especially when confronted with unpredictable loads (perturbations), such as lifting objects of varying weights or securely managing inertial properties when we slip. Central to this compensation mechanism is the transcortical long‐latency reflex (LLR), discovered over five decades ago (Hammond, 1956). Occurring 50–150 ms after limb displacement, the LLR represents a fast feedback pre‐voluntary response, bridging the gap between the rapid short‐latency reflex (SLR) and the slower voluntary reactions (Marsden et al., 1983). Unlike the short‐latency reflex (SLR), which relies solely on spinal networks and group I afferents (Lourenço et al., 2006), the LLR integrates group I and group II afferents through both spinal and supraspinal circuits (Schuurmans et al., 2009), engaging distributed circuitry like the premotor cortex and basal ganglia (Shadmehr & Krakauer, 2008). This versatility extends to integrating sensory information across multiple muscles, a key for adapting to dynamic environments during human movement performance (Franklin et al., 2003). Nearly every athlete has experienced an LLR when they step off of a curb and experience the “apparent twisted ankle” or lift a weight that is “heavier than expected” inducing a natural perturbation to the neuromuscular control system. Naturally occurring unexpected perturbation responses to exercise may be distinct among males and females during human performance.

Incorporating sex as a biological variable in exercise induced perturbation research is needed because of known neural sexual dimorphism. Studies support larger cortical areas (Luders et al., 2006) and higher gray‐white matter composition (Allen et al., 2003) in females than males, but findings vary based on methods used to assess anatomical and morphological differences (Sacher et al., 2013). In movement control, sex‐specific differences remain underexplored. Females typically exhibit higher levels of muscle co‐activation, resulting in lower spinal H‐reflex excitability compared to males (Mendonca et al., 2020), along with greater variability in co‐activation (Casamento‐Moran et al., 2017). Lower musculotendinous stiffness with greater compliance in females (Blackburn et al., 2008), may delay spindle excitation and reduce mechanical coupling between the muscle and spindle (Rack et al., 1983). Along with fluctuating presynaptic inhibition level (Hoffman et al., 2018), this could lead to diminished muscle spindle sensitivity and, consequently, a lower stretch reflex amplitude compared to males (Blackburn et al., 2008). Hormonal fluctuations, particularly across the menstrual cycle, can further alter these reflex responses (Casey et al., 2014). These sex‐based differences in neuromuscular physiology suggest distinct strategies when responding to unexpected perturbations during exercise training.

This study focuses on the upper extremity visuomotor tracking task, a commonly studied behavior in skilled movement control research (Masson et al., 1995; Miall et al., 2001). Males typically show better visuomotor tracking accuracy than females (Carey et al., 1994), although some of the previous research on the matter has disputed these differences (Peters et al., 1990) or stated a female advantage in fine motor skills (Hall & Kimura, 1995; Nicholson & Kimura, 1996). This inconsistency highlights the complexity of how sex may influence movement performance across different contexts. However, sex‐specific differences in fast feedback responses to unexpected perturbations, when there is an error, have yet to be elucidated. It was shown that females are generally slower but more precise than males (Barral & Debû, 2004), reflecting potential distinct strategies used by males and females.

Despite advances in understanding interhemispheric communication during voluntary movements, significant gaps also remain in our knowledge of how perturbation‐induced responses in one exercising limb may prime movement on the contralateral side of the body. Cross‐transcortical contralateral communication during unilateral movements refers to the exchange of information and coordination between different regions of the cerebral cortex involved in movement processing (Ames & Churchland, 2019; Ocklenburg & Guo, 2024; van der Knaap & van der Ham, 2011). There is evidence that when both arms contribute to achieving a task goal, reflex responses are bilaterally elicited in response to unilateral perturbations (Mutha & Sainburg, 2009). No previous study, to our knowledge, has examined if a nonparticipating relaxed contralateral arm experiences a detectable reflexive response when an exercise‐induced perturbation is delivered to a unilateral actively exercising arm, demonstrating cross‐transcortical communication. Importantly, whether this response is modulated in both males and females warrants this investigation.

The overall goal of this study was to investigate sex differences in visuomotor tracking accuracy in response to unexpected perturbations during an elbow flexion movement task. We explored how varying resistance and velocity levels would modulate muscle activity before and after perturbation during exercise, and we determined whether a unilateral upper limb perturbation would evoke a corresponding LLR in the contralateral, nonparticipating arm, indicating cross‐transcortical communication. We hypothesized that males would exhibit greater tracking accuracy and a higher rate of angular change than females during visuomotor tracking with unexpected perturbations; that perturbation responses would be greater at higher resistance levels as compared to lower resistance levels; and that temporally aligned electromyographic activity would be present in the contralateral limb of an exercising unilateral limb during the LLR (50–150 ms). Collectively, we expect that females will show distinct differences in triggered responses and suggest that skill exercise training will vary based on sex.

## 2 MATERIALS AND METHODS

### 2.1 Subjects and instrumentation

Forty healthy participants (20 males and 20 females) were recruited for the study. Participant demographics and anthropometric data are summarized in Table 1. Males had significantly higher values for weight, height, intracellular water (ICW), extracellular water (ECW), total body water (TBW), lean body mass (LBM), skeletal muscle mass (SMM), body mass index (BMI), basal metabolic rate (BMR), and MVC torque production, while females showed higher extracellular to intracellular water ratio (ECW/ICW). No significant sex differences were found for age, body fat mass (BFM), percent body fat (PBF), or visceral fat area (VFA). The female group mean (sd) NIH Cognitive Composite Scores (NIH CCS) were 124 (7.9), while the male group NIH CCS were 123 (7.4). All participants underwent screening to ensure the absence of orthopedic, neuromuscular, or neurological deficits or disorders. Prior to participation, all participants provided informed consent. This research protocol was prospectively approved by the University of Iowa's Human Subjects Review Board and complied with the Declaration of Helsinki.

**TABLE 1:** Participants' demographic and anthropometric data for each sex.

	Female	Male
	(*n* = 20)	(*n* = 20)
Age	25.15 ± 4.59	28.35 ± 6.2
Weight (kg)	67.32 ± 14.02	83.11 ± 16.5
Height (cm)	170.9 ± 6.87	176. 45 ± 6.9
ICW	51.72 ± 6.73	69.67 ± 13.4
ECW	30.85 ± 3.92	40.16 ± 7. 39
ECW/ICW	0.6 ± 0.01	0.58 ± 0.01
TBW	82.56 ± 10.65	109.92 ± 20.75
BFM	35.39 ± 23.5	32.88 ± 16.88
LBM	113.03 ± 14.72	150.33 ± 28.87
SMM	63.05 ± 8.77	86.44 ± 17.46
PBF	22.48 ± 8.96	17.43 ± 7
BMI	23.05 ± 4.68	26.55 ± 4.28
VFA	67.4 ± 50.36	60.48 ± 36.88
BMR	1477.35 ± 144.26	1842.95 ± 282.67
MVC (N·m)	39.2 ± 17.35	66.97 ± 19.91

*Note*: Values are mean ± standard deviation.

Abbreviations: BFM, body fat mass; BMI, body mass index; BMR, basal metabolic rate; ECW/ICW, ratio of ECW to ICW; ECW, extracellular water; ICW, intracellular water; LBM, lean body mass; PBF, percent body fat; SMM, skeletal muscle mass; TBW, total body water; VFA, visceral fat area.

This study analyzes secondary outcomes from a previous report (Dubey et al., 2025). Briefly, a custom‐built Neuromuscular Therapeutic Training System (NTTS) was employed which featured a sophisticated gear, magnetic brake, and movement sensors connected to the participants' left upper extremity. The left arm, which served as the nondominant limb, was used to perform all movement tasks. To ensure stability and accuracy, left arm and forearm were securely positioned within fitted cuffs, and the hands were firmly strapped to the device. The right arm remained at rest in a mirror‐matched posture to the left arm, but it did not perform any movements. The right was intentionally left unrestrained to avoid drawing participants' attention to it or affording any resistance to facilitate voluntary activation. Movements of the left arm flexion and extension caused rotation of a shaft connected to a potentiometer to accurately measure angular displacement. Additionally, an electromagnetic braking system was utilized to control the resistance experienced by the user, with adjustments made based on each participant's maximum voluntary contraction (MVC) of the left triceps. The braking system was directly managed by a microcomputer through digital‐to‐analog input, with all operations governed by customized software. To provide visual feedback, the system displayed real‐time information regarding elbow displacement (ranging from 0° to 40°) on a computer screen positioned in front of the participant. Calibration of the device ensured excellent linearity, repeatability, and minimal hysteresis of the braking and potentiometer system, maintaining accuracy within less than 0.5% of full scale. The relationship between voltage (X) and angular displacement (Y) was precisely determined, with 1‐degree change corresponding to approximately 0.02 volts.

### 2.2 Experimental setup

**FIGURE 1:**
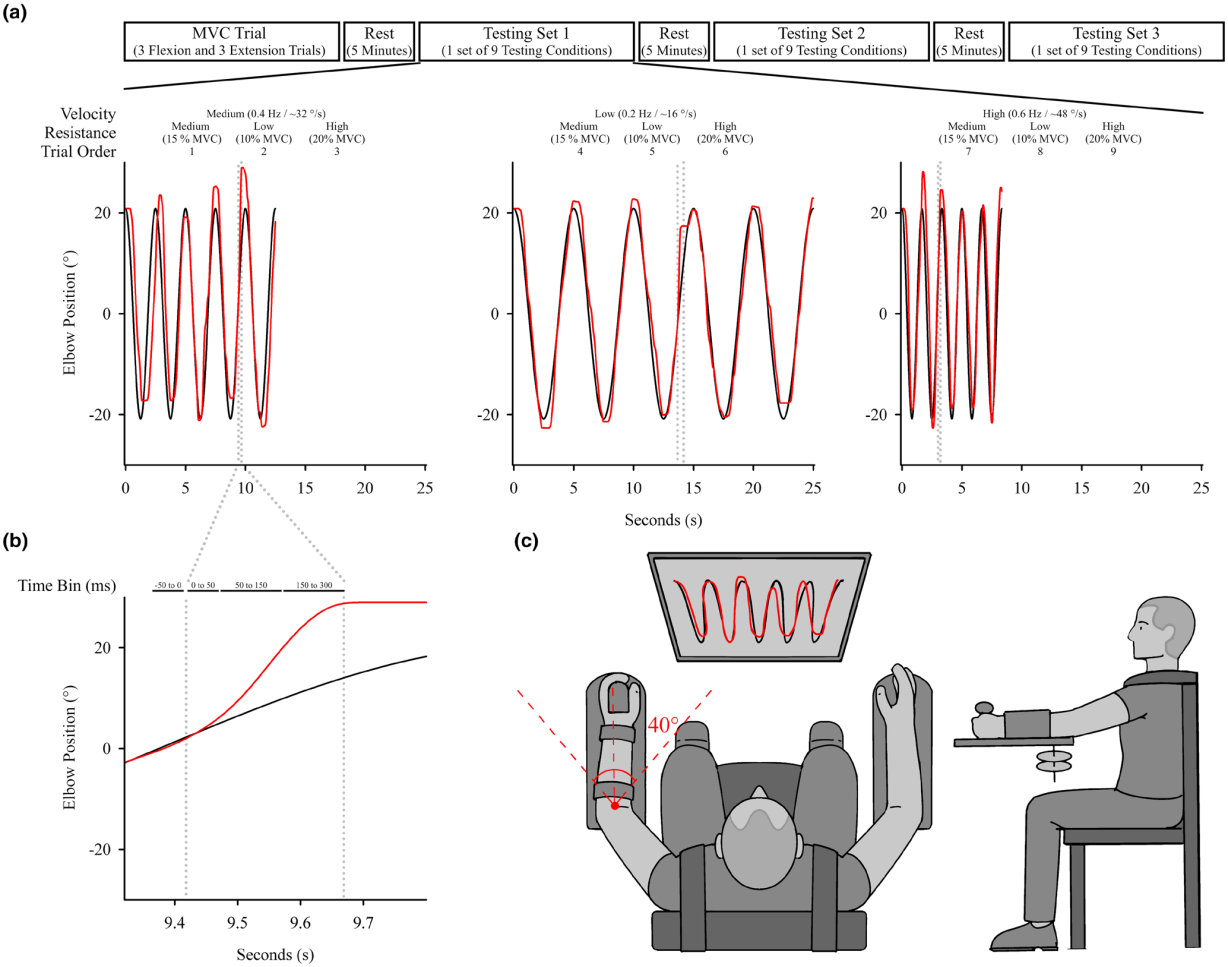
Experimental setup. (a) Schematic of the study design with a representative example of the movement control task at the medium, low, and high velocities. The black line represents the target line generated by the software and the red line represents the user's line obtained from the participant's left arm position. The vertical gray line indicates the start and end of the unexpected perturbation. (b) A representative example of performance during a perturbation for each time bin subset: −50 to 0 ms (pre‐perturbed), 0–50 ms (SLR), 50–150 ms (LLR), and 150–300 ms (VR). (c) Schematic of movement control device and participant positioning during the movement control task. MVC, maximal voluntary contraction.

The experimental protocol involved a single session utilizing the Neuromuscular Therapeutic Training System (NTTS) (see Figure 1c), which facilitated the implementation of nine distinct exercise testing conditions. These conditions combined various movement speeds (0.2, 0.4, and 0.6 Hz) with different levels of brake resistance (10%, 15%, and 20% of the participant's left triceps MVC). A standardized 30‐s rest interval separated each testing condition to minimize fatigue effects. The order of resistance and speed conditions was fixed across participants to maintain a standardized progression of task difficulty and to control for learning and carryover effects. This approach ensured that all participants experienced the same sequence of motor demands, facilitating direct comparisons of performance and neuromuscular responses across conditions. The condition order was as follows: medium speed and medium resistance, medium speed and low resistance, medium speed and high resistance, slow speed and medium resistance, slow speed and low resistance, slow speed and high resistance, high speed and medium resistance, high speed and low resistance, high speed and low resistance. Each participant went through each of these conditions three a total of three times (three attempts with each attempt involving the 9 conditions). Participants tracked a computer‐generated sinusoidal target line across five cycles (Figure 1a,b). Each cycle corresponded to a half‐period of sinusoid (T/2) of forearm extension and flexion. Despite variations in movement speeds, participants were instructed to maintain consistent body positions and precise control over elbow angular motion during the exercise. A second line indicating participant arm position was superimposed on the target line, providing real‐time visual feedback on performance accuracy. A single practice trial was given to familiarize the participant with the device and movement task. The practice and subsequent testing trials consisted of the participant completing five extension‐flexion cycles where a perturbation was randomly delivered during the flexion phase (half‐period) of the trial. After the practice trial, participants completed three sets of nine trial conditions with a 5‐min rest period between sets. A task performance error score was presented at the end of each set for participant feedback on task performance.

### 2.3 Performance variables and EMG

Performance variables, including absolute error (AE) and peak user rate, were measured during the visuomotor exercise task to assess the magnitude and rate of deviation from the intended trajectory. AE represented the magnitude of deviation between the target trajectory and the participant's actual movement during the visuomotor tracking task. It quantified the distance between corresponding points on the target and user traces, irrespective of the direction of the deviation. Peak AE for each cycle denoted the maximum error magnitude within each flexion extension cycle (perturbed and non‐perturbed). Peak AE was also calculated for each time bin (−50 to 0 ms pre‐perturbation; 0–50 ms (SLR); 50–150 ms (LLR); 150–300 ms volitional response (VR) post‐perturbation). The user rate, defined as the slope of the user trace, reflected the instantaneous rate of change in movement or the speed at which the participant's trajectory diverged from the target at the time of perturbation. Surface electromyography (EMG) signals were collected from specific muscles of both upper extremities to elucidate muscular activity patterns. The selected muscles for EMG recording included the left triceps long head, left triceps lateral head, left biceps, right triceps long head, right triceps lateral head, and right biceps. Prior to electrode placement, the skin was cleansed with alcohol to ensure optimal contact and signal quality. Wireless EMG sensors (Delsys Trigno, Natick, MA) were placed over each muscle belly, according to SENIAM guidelines (Hermens et al., 2000). Lateral triceps head electrodes were placed at 50% on the line between the posterior crista of the acromion and the olecranon at 2 finger widths lateral to the line, in the direction of the line between the posterior crista of the acromion and the olecranon process. Triceps long head electrodes were placed at 50% on the line between the posterior crista of the acromion and the olecranon at 2 finger widths medial to the line, in the direction of the line between the posterior crista of the acromion and the olecranon. Biceps electrodes were placed on the line between the medial acromion and the fossa cubit at 1/3 from the fossa cubit, in the direction of the line between the acromion and the fossa cubit.

All EMG sampling, both during MVC and the visuomotor exercise task, was conducted using LabVIEW software (National Instruments; Austin, TX). EMG signals were collected with a bandwidth of 20–450 Hz, a common mode rejection ratio of >80 dB, 16‐bit resolution, and a sampling rate of 2000 Hz. All EMG data was processed offline using the root mean square (RMS) method, averaged over 10 ms intervals. To establish a baseline for normalization of the left arm EMG, participants performed three maximum voluntary isometric contractions in both elbow flexion and extension of the left arm, with verbal encouragement provided during each contraction. EMG activity from the right arm was calculated at the task performance time intervals defined by the left arm. EMG activity for each muscle was normalized to the peak pre‐perturbation muscle activity (−50 to 0 ms) of the right arm.

### 2.4 Perturbation and data collection

The perturbation paradigm involved the unexpected and random removal of brake resistance for a pre‐determined duration, strategically timed to coincide with the early elbow flexion phase (i.e., 10° of elbow flexion) of the movement to induce stretch to the triceps. A perturbation was inserted randomly within the 2nd to 5th sinusoid cycles (half‐periods) for both practice and testing trials. This randomization prevented participants from predicting the timing of the perturbation within each trial, thereby preserving its unexpected nature. The perturbation duration was scaled to match the movement speed, lasting 500 ms, 250 ms, and 200 ms for slow (0.2 Hz), medium (0.4 Hz), and fast (0.6 Hz) target frequencies, respectively. The percentage of muscle activation during perturbations was calculated using the following formula: LLR% = (LLR EMG activity – Pre‐perturbed EMG activity)/Pre‐perturbed EMG activity × 100.

### 2.5 Statistical analysis

We conducted data analysis using R Statistical Software (R Core Team (2022). R: A language and environment for statistical computing. Vienna, Austria). We elected to perform multiple two‐way analysis of variance (ANOVA) with a mixed model to address specific research questions. We did not use a comprehensive multifactorial ANOVA given the number of independent variables. Therefore, we analyzed differences between the sexes at each time bin before (−50 to 0 ms) and after the perturbation (0–50 ms (SLR), 50–150 ms (LLR), and 150–300 ms) (VR, Volitional Reaction). Using this model, we evaluated differences in peak AE, peak user rate, muscle activity (left and right biceps, triceps lateral head and triceps long head), and the ratio of the antagonist (triceps) to agonist (biceps) (TtB ratio). Additionally, we evaluated these dependent variables (peak AE, peak user rate, muscle activity, and TtB ratio) across each time bin by comparing the perturbed cycle to the non‐perturbed cycles for each sex separately (Time bin vs. Cycle). Finally, we compared the influence of task resistance on EMG activity. A two‐way mixed model ANOVA was used to assess change in EMG activity between the sexes and time bins for only the perturbed cycle at the low and high resistance levels, separately (Time Bin vs. Sex). We also compared the differences of sex and resistance level on EMG activity for only the perturbation cycle at each time bin, separately (Sex vs. Resistance). All assumptions were evaluated prior to each test using the Shapiro–Wilk test for normality of residuals and Mauchly's test for sphericity. A Greenhouse–Geisser correction (GG) was applied in all instances when the sphericity assumption was violated. Significant omnibus effects and interactions were followed by Tukey's HSD post‐hoc tests to perform pairwise comparisons while controlling the family‐wise error rate for those comparisons. We report F, df (including corrected df when applicable), *p*, and partial *η* ^2^ for omnibus tests, and adjusted *p* values for post‐hoc comparisons. The significance level was set at *p* < 0.05. All reported values in mean ± 1 standard deviation (SD).

## 3 RESULTS

### 3.1 Performance assessment

**FIGURE 2:**
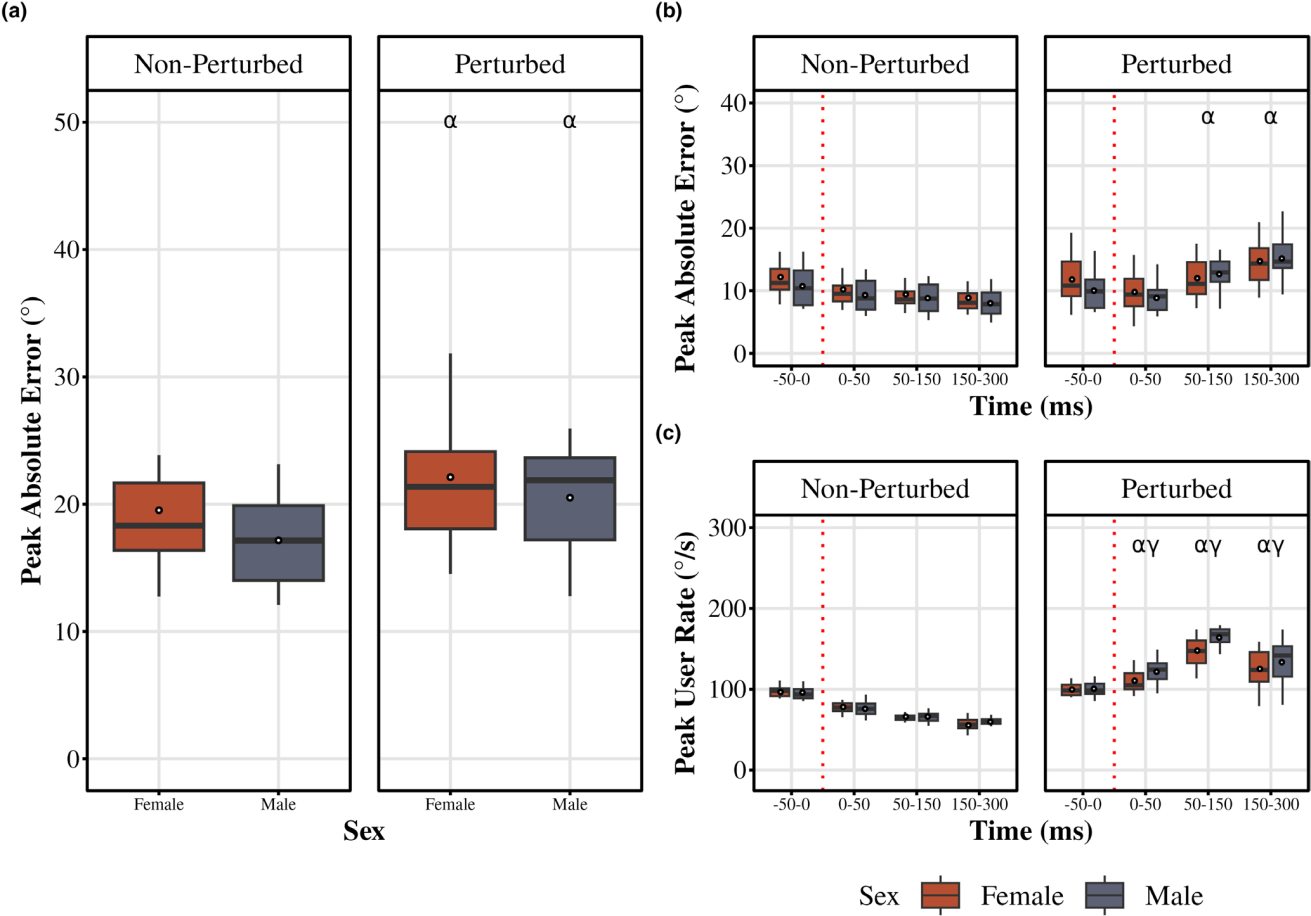
Tracking accuracy and user rate across time and cycle types. (a) Peak absolute error (AE) in degrees (°) during the non‐perturbed (left) and perturbed (right) cycles for females and males. (b) Peak AE across time bins (−50 to 0 ms (pre‐perturbed), 0–50 ms (SLR), 50–150 ms (LLR), and 150–300 ms) (VR) during the non‐perturbed (left) and perturbed (right) cycles for females and males. (c) Peak user rate (slope) across time bins during the non‐perturbed (left) and perturbed (right) cycles for females and males. Red dotted line reflects the delivery of the perturbation in the perturbed cycle and point where a perturbation would have occurred in the non‐perturbed cycle. Boxplots display the median (black line), interquartile range (box), and range (whiskers); white dots represent the mean. α indicates a difference between the perturbed and non‐perturbed cycle (females and males not pooled); β indicates difference between sexes within during the perturbed cycle at the indicated time bin; γ indicates difference between the indicated time bin and the −50 to 0 ms (pre‐perturbed) time bin (for both females and males).

We assessed the absolute error (AE) scores with and without perturbation to ensure the delivery of the perturbation for both sexes (Figure 2a). There was a main effect of cycle on the peak AE (F (1, 38) = 80.77, *p* < 0.0001, partial *η* ^2^ = 0.68). Peak AE was higher during the perturbed cycle for both males and females (*p* < 0.0001), verifying the perturbation was delivered. However, there was no difference between males and females during both cycles with (*p* = 0.30) and without perturbation (*p* = 0.12).

Next, we evaluated how the peak AE changes across time bins (−50 to 0 ms before the perturbation, 0–50 ms (SLR), 50–150 ms (LLR), and 150–300 ms (VR) after the perturbation) for each sex (Figure 2b). There was an interaction between time bin and sex during the perturbed cycle (*F* (1.36, 51.9) = 3.65, *p* = 0.049, partial *η* ^2^ = 0.088; GG). Peak AE decreased during 0–50 ms (SLR) post‐perturbation (*p* = 0.008), and then AE increased in 50–100 ms (LLR, *p* = 0.002) and 150–300 ms (VR, *p* = 0.0001) relative to the non‐perturbed condition. Males increased AE in 50–100 ms (LLR) and 150–300 ms (VR) (all *p* < 0.0001). However, there was no difference between males and females through all time bins. There was a higher peak of AE during 50–150 ms (LLR) and 150–300 ms (VR) when compared to the non‐perturbed cycle for both sexes (all, *p* < 0.004). There was no difference in peak AE during the −50 to 0 ms and 0–50 ms (SLR) time bins between the perturbed and non‐perturbed cycles for both sexes.

As for the user rate, there was no interaction between time bin and sex during the perturbed cycle (*F* (1.8, 68.56) = 1.57, *p* = 0.22, partial *η* ^2^ = 0.04; GG; Figure 2c), but there was a main time effect (*p* < 0.001). Peak user rate was higher in the 0–50 ms, 50–150 ms, and 150–300 ms windows (*p* < 0.001) as compared to the non‐perturbed condition. There was a significant interaction in peak user rate for both females (*F* (1.8, 34.3) = 122.4, *p* < 0.001, partial *η* ^2^ = 0.04; GG) and males (*F* (1.8, 34.3) = 122.4, *p* < 0.001, partial *η* ^2^ = 0.04; GG) between time and cycle. While there was no difference between peak user rate in the −50 to 0 ms before a perturbation between the perturbed and non‐perturbed cycles for either females (*p* = 0.43) or males (*p* = 0.27), both females and males had higher peak user rates during the perturbation cycle for the 0–50 ms, 50–150 ms, and 150–300 ms time windows (*p* < 0.001).

### 3.2 EMG response to the unexpected perturbation

**FIGURE 3:**
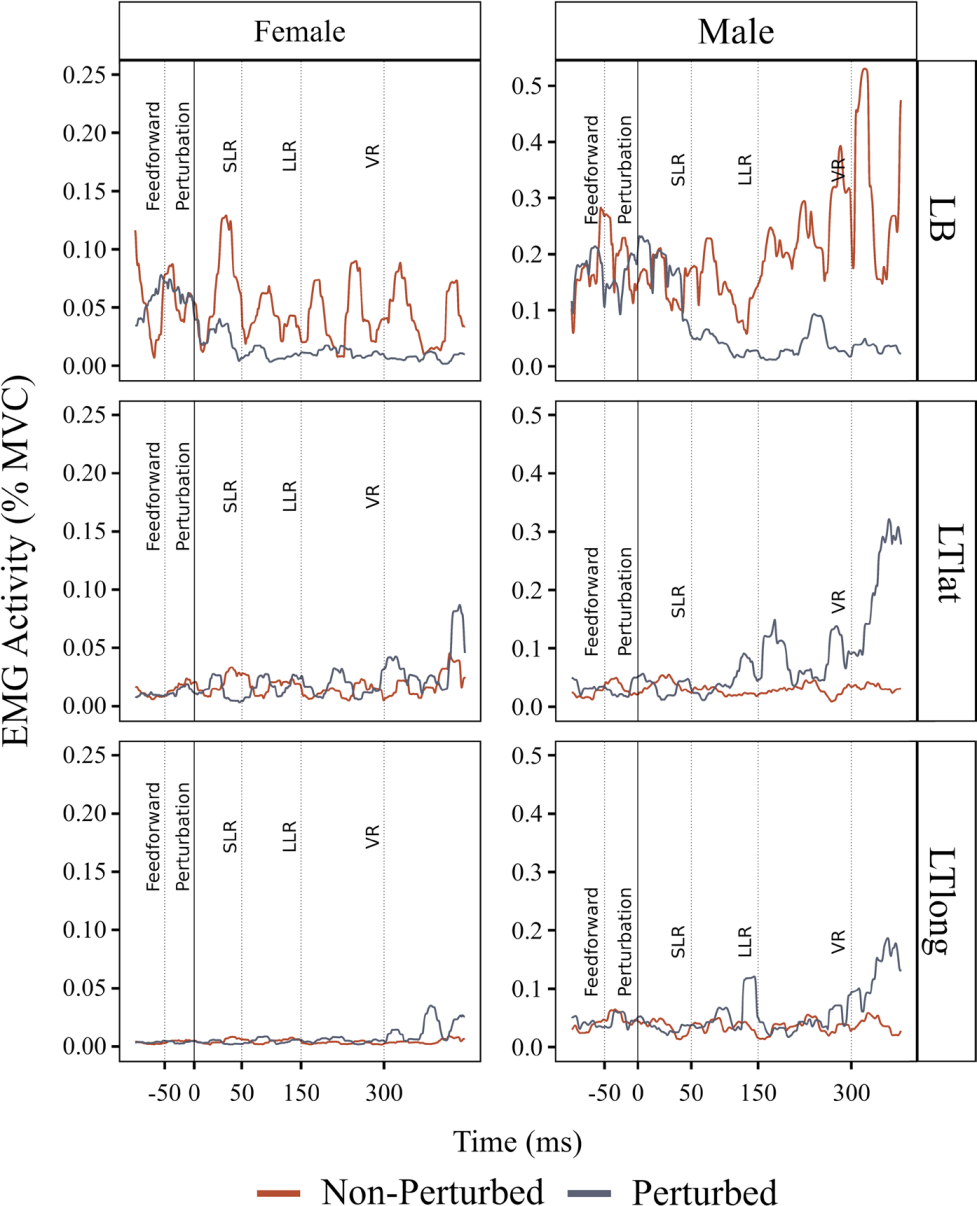
An example of a root mean square (RMS) transformed EMG signal during a perturbed and non‐perturbed cycle. A representative female (left) and male (right) example of RMS transformed EMG signal of the left biceps (LB, top), triceps lateral head (LTlat, middle), left triceps long head (LTlong, bottom) during the movement control task. Time periods used for EMG analysis: Feedforward: −50 to 0 ms prior to perturbation; Short‐Latency Reflex (SLR): 0–50 ms following perturbation; Long‐Latency Reflex (LLR): 50 to 150 ms following perturbation, Voluntary Reaction (VR): 150–300 ms following perturbation.

Figure 3 illustrates representative EMG activity in the left biceps (LB), lateral head of the triceps (LTlat), and long head of the triceps (LTlong) for one male and one female participant. All EMG values were normalized to their respective maximum voluntary contraction (MVC) values.

**FIGURE 4:**
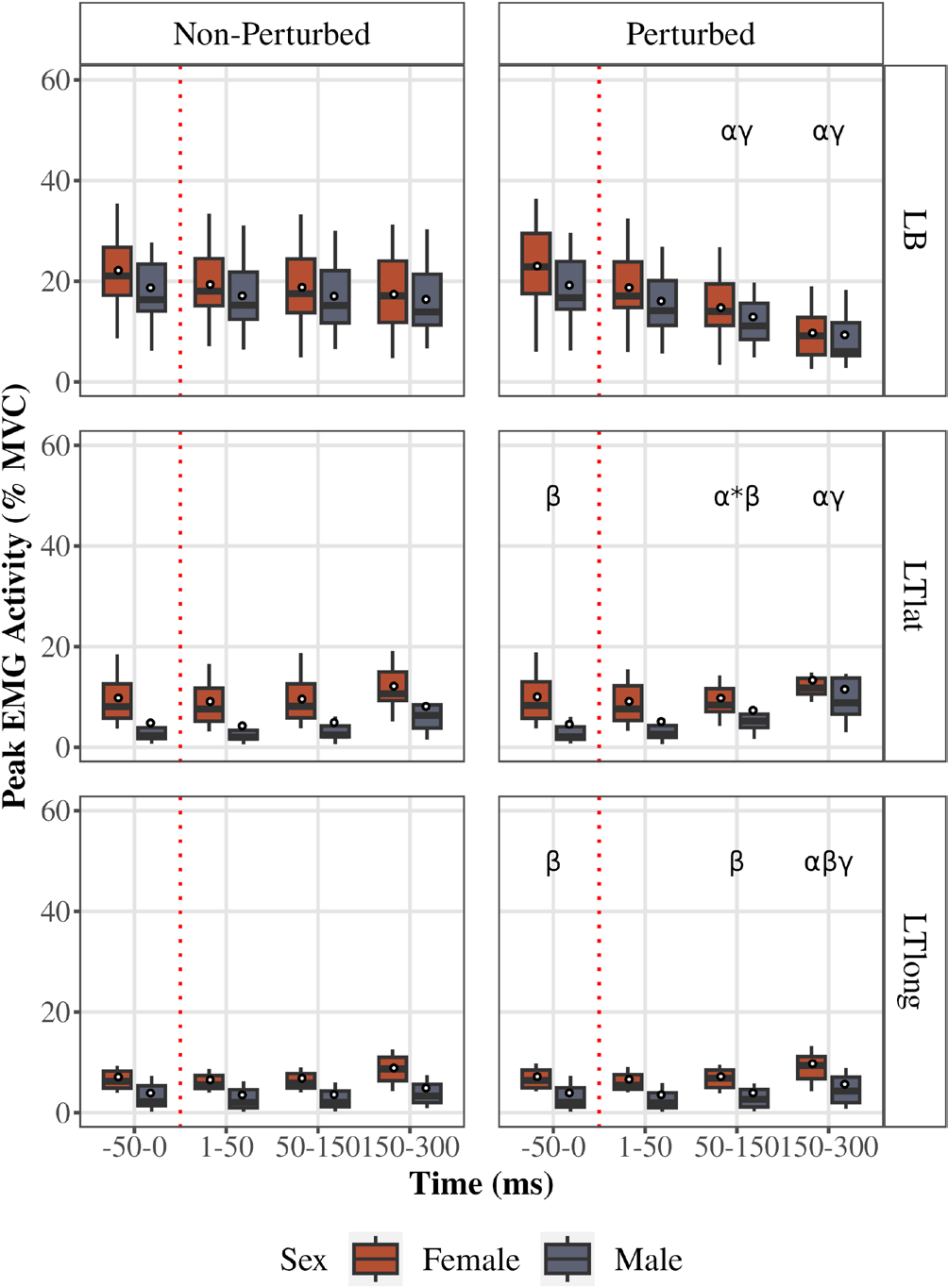
EMG activity for each sex during a perturbed and non‐perturbed cycle. Peak EMG activity (normalized to MVC) of the perturbed (right) and non‐perturbed (left) cycles for Left biceps (LB, top), Left triceps lateral head (LTlat, middle), and Left triceps long head (LTlong, bottom) at −50 to 0 ms before the perturbation and 50–150 ms (LLR) and 150–300 ms (VR) after the perturbation. Red dotted line reflects the delivery of the perturbation in the perturbed cycle and the point where a perturbation would have occurred in the non‐perturbed cycle. Boxplots display the median (black line), interquartile range (box), and range (whiskers); white dots represent the mean. α indicates a difference between the perturbed and non‐perturbed cycle (females and males not pooled); β indicates difference between sexes during the perturbed cycle at the indicated time bin; γ–indicates difference between the indicated time bin and the −50 to 0 ms (pre‐perturbed) time bin (for both females and males); * indicates the α or γ annotation applies to only males.

There was an interaction of cycle type × time bin for the LB in females (*F* (1.16, 22.13) = 74.79, *p* < 0.0001, partial *η* ^2^ = 0.79; GG) and males (*F* (1.36, 25.85) = 96.88, *p* < 0.0001, partial *η* ^2^ = 0.84; GG) (Figure 4). Both sexes showed lower LB activity during 50–150 ms (LLR) and 150–300 ms (VR) time bins in the perturbed cycle compared to non‐perturbed (all *p* < 0.0001). 50–150 ms (LLR) and 150–300 ms (VR) activity was lower than pre‐perturbed in the perturbed cycles for both sexes (all *p* < 0.0001). However, there were no differences in LB activity between males and females during all time bins.

There was a main effect of time bin for females for the LTlat (*F* (1.35, 25.64) = 27.21, *p* < 0.0001, partial *η* ^2^ = 0.59; GG; Figure 4). 150–300 ms (VR) activity was higher in the perturbed cycle than non‐perturbed in females (*p* = 0.007). Females' LTlat activity was also higher in 150–300 ms (VR) compared to pre‐perturbed and 50–150 ms (LLR) (*p* < 0.0001), but 50–150 ms (LLR) was not different from pre‐perturbed. There was an interaction of cycle type × time bin for males (*F* (1.56, 29.73) = 16.13, *p* < 0.0001, partial *η* ^2^ = 0.46; GG). Males' 50–150 ms (LLR) (*p* = 0.0021) and 150–300 ms (VR) (*p* < 0.0001) activities were higher in the perturbed cycle. Males' 150–300 ms (VR) activity was higher than pre‐perturbed and 50–150 ms (LLR) (*p* < 0.0001). In the perturbed cycle, there was a main effect of sex (*F* (1, 38) = 6.286, *p* = 0.017, partial *η* ^2^ = 0.14). Females showed higher −50 to 0 ms (pre‐perturbed) and 50–150 ms (LLR) activity than males (*p* = 0.002, *p* = 0.043, respectively).

There was a main effect of time bin for females for the LTlong (F (1.15, 21.9) = 23.91, *p* < 0.0001, partial *η* ^2^ = 0.56; GG; Figure 4). 150–300 ms (VR) activity was higher in perturbed cycle than non‐perturbed in females (*p* = 0.004). In perturbed cycle, females' LTlong activity was higher in 150–300 ms (VR) bin compared to pre‐perturbed and 50–150 ms (LLR) (*p* < 0.0001), but 50–150 ms (LLR) was not different from pre‐perturbed. There was an interaction of cycle type × time bin for males (*F* (1.86, 35.38) = 5.26, *p* = 0.011, partial *η* ^2^ = 0.22; GG). Males' 50–150 ms (VR) activity was higher in the perturbed cycle (*p* = 0.0006). Males' 150–300 ms (VR) activity was higher than pre‐perturbed and 50–150 ms (LLR) (*p* < 0.0001). In the perturbed cycle, there was a main effect of sex (*F* (1, 38) = 8.25, *p* = 0.007, partial *η* ^2^ = 0.18). Females show higher pre‐perturbation, LLR and VR activity than males (*p* = 0.017, *p* = 0.016, and *p* = 0.003, respectively).

### 3.3 The influence of exercise resistance level on the EMG activity

#### 3.3.1 LB (Figure 5)

**FIGURE 5:**
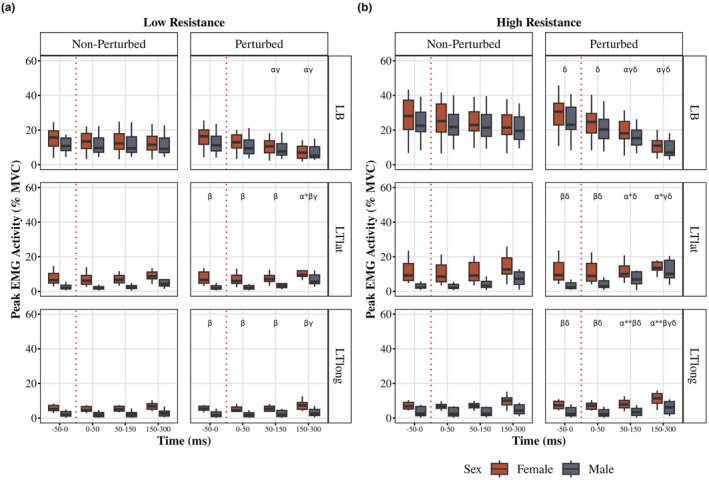
The influence of resistance level on the EMG activity of the left arm. EMG activity (normalized to MVC) during the low resistance (10% MVC torque, a) and high resistance (20% MVC torque, b) trials. The perturbed (right) and non‐perturbed (left) cycles for the Left biceps (LB, top), Left triceps lateral head (LTlat, middle), and Left triceps long head (LTlong, bottom) are shown across the −50 to 0 ms (pre‐perturbed), 50–150 ms (LLR), and 150–300 ms (VR) time bins for each sex. Boxplots display the median (black line), interquartile range (box), and range (whiskers); white dots represent the mean. α indicates a difference between the perturbed and non‐perturbed cycle (females and males not pooled); β indicates difference between sexes during the perturbed cycle at the indicated time bin; γ indicates difference between the indicated time bin and the −50 to 0 ms (pre‐perturbed) time bin (females and males not pooled); δ indicates a difference between the low and high resistance in the perturbed cycle (for both females and males); * indicates the α or γ annotation applies to only males; ** indicates the α or γ annotation applies to only females.

For females in the perturbed cycle, there was an interaction of time bin × resistance level (*F* (1.1, 20.96) = 33.34, *p* < 0.0001, partial *η* ^2^ = 0.64; GG). Females' LB activity was higher within high resistance conditions (pre‐perturbed and 50–150 ms (LLR) *p* < 0.0001, 150–300 ms (VR) *p* = 0.006). Low resistance condition led to 50–150 ms (LLR) inhibition compared to pre‐perturbed (*p* = 0.0005), but 150–300 ms (VR) was not different from 50 to 150 ms (LLR) (*p* = 0.1) in females. High resistance condition in females led to subsequent 50–150 ms (LLR) and 150–300 ms (VR) inhibition (all *p* < 0.0001). Similarly, for males in the perturbed cycle, there was an interaction of time bin × resistance level (*F* (1.56, 29.71) = 111.91, *p* < 0.0001, partial *η* ^2^ = 0.85; GG). Males' LB activity was higher within high resistance conditions (pre‐perturbed and 50–150 ms (LLR) *p* < 0.0001, VR *p* = 0.01). Low resistance condition led to 50–150 ms (LLR) inhibition compared to pre‐perturbed (p < 0.0001), but 150–300 ms (VR) was not different from 50 to 150 ms (LLR) (*p* = 0.09) in males. High resistance condition in males led to subsequent 50–150 ms (LLR) and 150–300 ms (VR) inhibition (all p < 0.0001). 50–150 ms (LLR) activity in low resistance condition was lower in perturbed cycle (main effect of cycle type *F* (1, 38) = 99.05, *p* < 0.0001, partial *η* ^2^ = 0.72) for both males and females (both p < 0.0001). 150–50 ms (VR) activity in low resistance condition was lower in perturbed cycle (main effect of cycle type *F* (1, 38) = 97.88, *p* < 0.0001, partial *η* ^2^ = 0.72) for both males and females (both p < 0.0001). Females' 50–150 ms (LLR) and 150–300 ms (VR) activities in both cycle types were not different from males. The findings in the high resistance condition were similar.

#### 3.3.2 LTlat (Figure 5)

For females in the perturbed cycle, there was an interaction of time bin × resistance level (*F* (1.47, 28.02) = 7.1, *p* = 0.006, partial *η* ^2^ = 0.27; GG). Females' LTlat activity was higher within high resistance conditions (all *p* < 0.0001). Neither low nor high resistance condition led to increase in 50–150 ms (LLR) compared to pre‐perturbed, but 150–300 ms (VR) was higher from 50 to 150 ms (LLR) (*p* = 0.001 in low, p < 0.0001 in high resistance) in females. For males in the perturbed cycle, there was a main effect of time (*F* (1.09, 20.65) = 12.49, *p* = 0.0008, partial *η* ^2^ = 0.397; GG) and resistance level (*F* (1, 19) = 34.25, *p* < 0.0001, partial *η* ^2^ = 0.64). Males' LTlat activity was higher within high resistance conditions (pre‐perturbed *p* = 0.01, 50–150 ms (LLR) *p* = 0.0001, 150–300 ms (VR) *p* < 0.0001). Neither low nor high resistance conditions led to significant increase in 50–150 ms (LLR) compared to pre‐perturbed activity, but 150–300 ms (VR) was higher from 50 to 150 ms (LLR) (*p* = 0.031 in low, *p* = 0.0003 in high resistance) for males. Within pre‐perturbation time bin of low resistance conditions, females had higher activity than males in both cycles (main effect of sex (*F* (1, 38) = 10.73, *p* = 0.002, partial *η* ^2^ = 0.22)). Within LLR time bin of low resistance conditions, females had higher activity than males in both cycles (main effect of sex (*F* (1, 38) = 3.17, *p* < 0.0001, partial *η* ^2^ = 0.52)). Males LLR activity in the perturbed cycle of low resistance conditions was higher than non‐perturbed (*p* = 0.013). Within 50–150 ms (LLR) time bin of high resistance conditions, there was a main effect of sex (*F* (1, 38) = 6.89, *p* = 0.012, partial *η* ^2^ = 0.15) and cycle (*F* (1, 38) = 7.16, *p* = 0.01, partial *η* ^2^ = 0.16). Males 50‐150 ms (LLR) activity in the perturbed cycle of high resistance conditions was higher than non‐perturbed (*p* = 0.002). 150–300 ms (VR) activity after a perturbation was higher than non‐perturbed only in males within low (*p* = 0.04) and high resistance condition (*p* = 0.0004).

#### 3.3.3 LTlong (Figure 5)

For females in the perturbed cycle, there was an interaction of time bin × resistance level (*F* (1.46, 27.71) = 20.73, *p* < 0.0001 partial *η* ^2^ = 0.52; GG). Females' LTlong activity was higher within high resistance conditions (all *p* < 0.0005). Neither low nor high resistance condition did not lead to increase in 50–150 ms (LLR) compared to pre‐perturbed, but 150–300 ms (VR) was higher from LLR (*p* = 0.008 in low, *p* < 0.0001 in high resistance) in females. For males in the perturbed cycle, there was an interaction of time bin × resistance level (*F* (1.18, 22.36) = 5,56, *p* = 0.002, partial *η* ^2^ = 0.23; GG). Males' LTlong activity was higher within high resistance conditions (pre‐p *p* = 0.002, LLR *p* = 0.0009, VR *p* < 0.0001). Neither low nor high resistance conditions led to significant increase in 50–150 ms (LLR) compared to pre‐perturbed activity, but 150–300 ms (VR) was higher from 50 to 150 ms (LLR) (*p* = 0.008 in low, *p* < 0.0001 in high resistance). Within pre‐perturbation time bin of low resistance conditions, females had higher activity than males in both cycles (main effect of sex (*F* (1, 38) = 7.35, *p* = 0.001, partial *η* ^2^ = 0.02)). Within 150–50 ms (LLR) time bin of low resistance conditions, females had higher activity than males in both cycles (main effect of sex (*F* (1, 38) = 7.93, *p* = 0.0008, partial *η* ^2^ = 0.17)). 50–150 ms (LLR) activity in the perturbed cycle of low resistance conditions was not different from non‐perturbed for both sexes. Within 50–150 ms (LLR) time bin of high resistance conditions, there was a main effect of sex (*F* (1, 38) = 8.4, *p* = 0.0006, partial *η* ^2^ = 0.18) and cycle (*F* (1, 38) = 4.44, *p* = 0.01, partial *η* ^2^ = 0.11). Females 50–150 ms (LLR) activity in the perturbed cycle of high resistance conditions was higher than non‐perturbed (*p* = 0.029), males' 50–150 ms (LLR) did not differ across cycles. 150–300 ms (VR) activity after a perturbation was higher only in females within high resistance condition (*p* = 0.03).

### 3.4 Muscle activation

**FIGURE 6:**
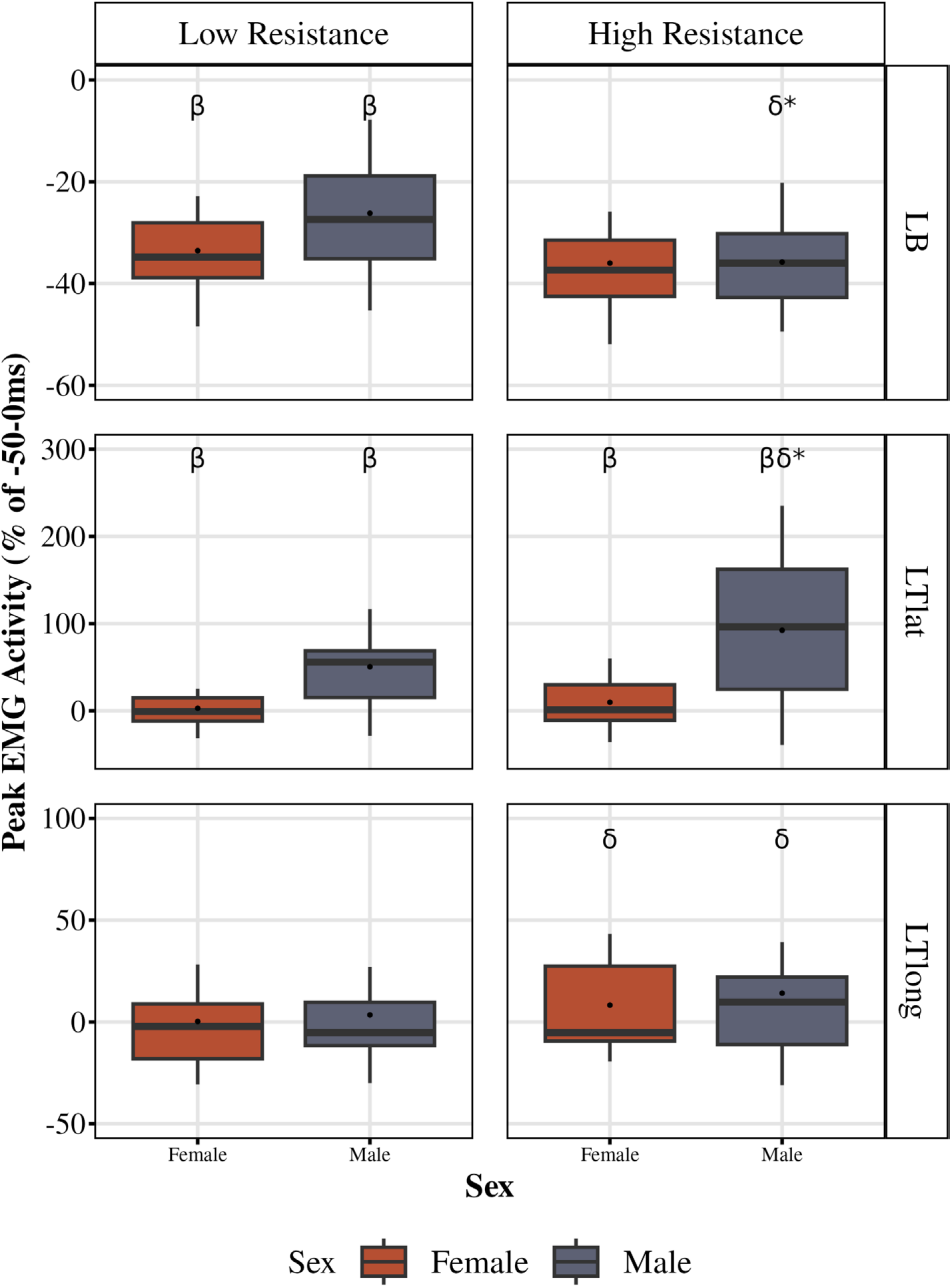
EMG activation during 50–150 ms (LLR) time bin in left biceps (LB), left triceps lateral head (LTlat), and left triceps long head (LTlong). The activation difference (as a percent) of the −50 to 0 ms (pre‐perturbed) time bin during the 50–150 ms (LLR) after the perturbation during the low resistant (10% MVC torque, a) and high resistance (20% MVC torque) for the Left biceps (LB, top), Left triceps lateral head (LTlat, middle), and Left triceps long head (LTlong, bottom). Percent activation was calculated as: [50–150 ms (LLR) – −50 to 0 ms (pre‐perturbed)]/[−50 to 0 ms (pre‐perturbed)] × 100%. Boxplots display the median (black line), interquartile range (box), and range (whiskers); white dots represent the mean. β indicates difference between sexes within the perturbed cycle; δ indicates a difference between the low and high resistance in the perturbed cycle; * indicates the β or δ annotation applies to only males.

In the left biceps (LB), during low resistance conditions, activation decreased (i.e., inhibition), with females showing a mean activation difference of −33.54 ± 2.32% and males −25.83 ± 2.51% of the pre‐perturbed time bin (−50 to 0 ms). Under high resistance conditions, both sexes showed further inhibition, with females at −35.99 ± 2.29% and males at −36.64 ± 2.30% (Figure 6). Females demonstrated more inhibition in low‐resistance conditions than males (main effect of sex (*F* (1, 38) = 4.98, *p* = 0.03, partial *η* ^2^ = 0.12)). For the resistance effect, there was a sex × resistance interaction (*F* (1, 36) = 7.67, *p* = 0.008, partial *η* ^2^ = 0.18). Females' LB inhibition was not different between low and high resistance conditions, while males displayed more inhibition in high resistance conditions (*p* < 0.0001).

In the left triceps lateral head (LTlat) under low resistance, females had an activation difference of 2.93 ± 5.46%, while males had 37.53 ± 9.39% of the pre‐perturbed time bin (−50 to 0 ms). Under high resistance, females had a mean activation difference of 8.07 ± 6.49%, and males had 59.64 ± 13.29% (Figure 6). Males had significantly higher activation % than females under both low resistance and high resistance conditions (main effect of sex (*F* (1, 38) = 19.86, *p* = 0.001, partial *η* ^2^ = 0.25) and resistance (*F* (1, 38) = 8.77, *p* = 0.005, partial *η* ^2^ = 0.19)). Females' LTlat activation % was not different between low and high resistance conditions, while males displayed more activation in high resistance conditions (*p* = 0.022).

In the left triceps long head (LTlong) under low resistance, females had an activation difference of 0.23 ± 5.20%, while males had −0.56 ± 6.71%. Under high resistance, females had a mean activation % of 7.54 ± 6.05%, and males had −5.27 ± 7.19% (Figure 6). There was a sex × resistance interaction (*F* (1, 38) = 7.43, *p* = 0.010, partial *η* ^2^ = 0.16) supporting that LTlong activation for Females was not consistent to Males across the low and high resistance conditions.

### 3.5 Co‐activation

**FIGURE 7:**
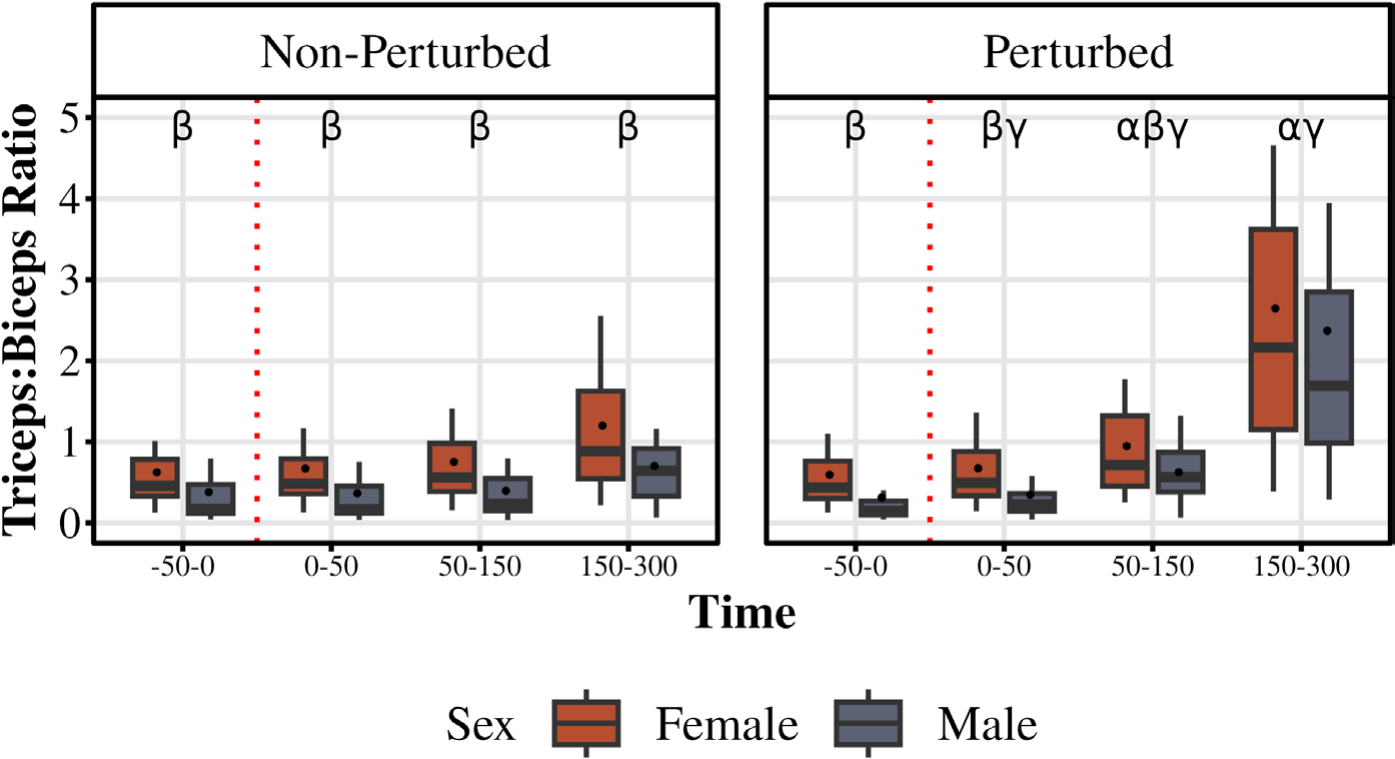
Triceps to biceps (TtB) ratio across time bins. The ratio of the left triceps (average of long head and lateral head of left triceps) to left biceps EMG activity during the perturbed (right) and non‐perturbed (left) for females and males. Boxplots display the median (black line), interquartile range (box), and range (whiskers); white dots represent the mean. α indicates a difference between the perturbed and non‐perturbed cycle (females and males not pooled); β indicates difference between sexes within during the perturbed cycle at the indicated time bin; γ indicates difference between the indicated time bin and the −50 to 0 ms (pre‐perturbed) time bin (for both females and males).

To assess the level of co‐activation between triceps and biceps muscles, we calculated the Triceps‐to‐Biceps (TtB) ratio (Figure 7). The mean EMG values for both tricep heads, normalized to their respective MVCs, were combined and divided by the normalized EMG of the biceps. There was a main effect of sex (*F* (1, 38) = 8.06, *p* = 0.02, partial *η* ^2^ = 0.13) and cycle (*F* (1, 38) = 4.67, *p* = 0.04, partial *η* ^2^ = 0.11) during the −50 to 0 ms (pre‐perturbed) time bin which was higher in females both in non‐perturbed (*p* = 0.03) and perturbed (*p* = 0.01) cycles compared to males. There was no difference between cycles within each sex. There was a main effect of sex (*F* (1, 38) = 7.33, *p* = 0.01, partial *η* ^2^ = 0.16) and cycle (*F* (1, 38) = 24.75, *p* = 0.014, partial *η* ^2^ = 0.39) during 50–150 ms (LLR) time bin where TtB was higher in females both in non‐perturbed (*p* = 0.008) and perturbed (*p* = 0.015) cycles compared to males. Both sexes had a higher TtB ratio during the 50–150 ms (LLR) time of perturbed cycle (female *p* = 0.0005, male *p* = 0.0025) compared to the non‐perturbed cycle. There was a main effect of cycle (*F* (1, 38) = 51.14, *p* < 0.0001, partial *η* ^2^ = 0.54) during 150–300 ms (VR) time bin. Both sexes had higher TtB ratio in 150–300 ms (VR) time of perturbed cycle (female *p* = 0.0005, male *p* = 0.0025) compared to the non‐perturbed cycle. 150–300 ms (VR) time bin TtB ratio was higher in females than males in non‐perturbed (*p* = 0.006), but not perturbed (*p* = 0.16) cycle. When analyzing the TtB ratio across time in the perturbed cycle, there was a main effect of time (*F* (1.03, 39.18) = 53.88, *p* < 0.0001, partial *η* ^2^ = 0.59, GG) where both females and males showed an increase in the TtB ratio during the 50–150 ms (LLR) time bin compared to −50 to 0 ms (pre‐perturbed) and 150–300 ms (VR) time bins (all *p* < 0.005).

### 3.6 Cross‐transcortical responses in the nonparticipating upper extremity

**FIGURE 8:**
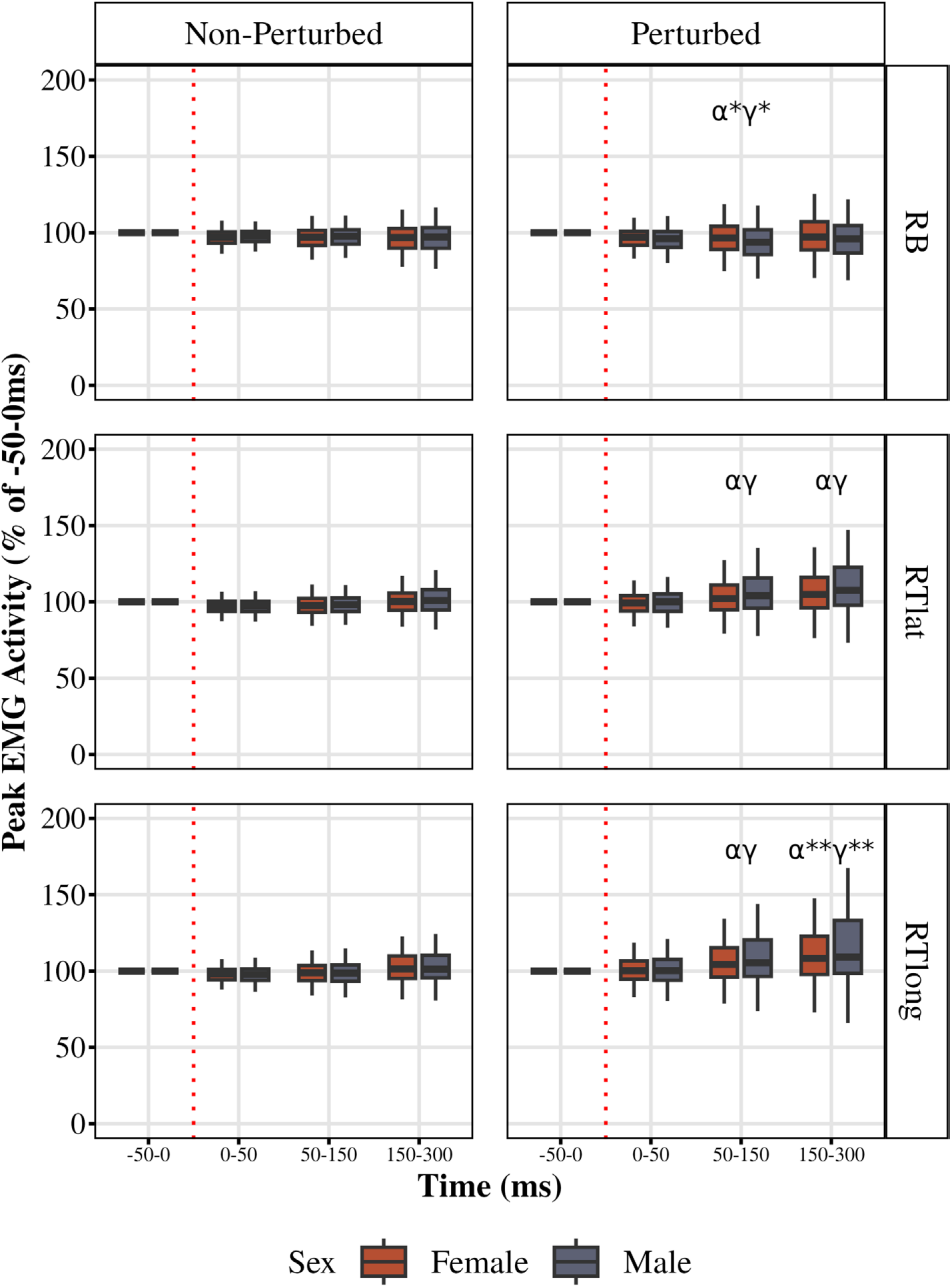
Electromyographic muscle activity of the right nonparticipating upper extremity. Peak muscle activity (normalized to the −50 to 0 ms (pre‐perturbed) time bin) of the nonparticipating arm during the perturbed (right) and non‐perturbed cycles at the −50 to 0 ms (pre‐perturbed), 0–50 ms (SLR), 50–150 ms (LLR), and 150–300 ms (VR) time bins in males and females for the Right biceps (RB, top), Right triceps lateral head (RTlat, middle), and Right triceps long head (RTlong, bottom). Boxplots display the median (black line), interquartile range (box), and range (whiskers). α indicates a difference between the perturbed and non‐perturbed cycle (females and males not pooled); β indicates difference between sexes within during the perturbed cycle at the indicated time bin; γ indicates difference between the indicated time bin and the −50 to 0 ms (pre‐perturbed) time bin (for both females and males); * indicates the α or γ annotation applies to only males; ** indicates the α or γ annotation applies to only females.

For the right biceps (RB, Figure 8), males had a main effect of time (*F* (1.74, 33) = 15.1, *p* < 0.001, partial *η* ^2^ = 0.44, GG). Males' post‐perturbation 50–150 ms (LLR) was lower in the perturbed cycle as compared to non‐perturbed (*p* = 0.029). Females' 50–150 ms (LLR) in the perturbed cycle was not different from non‐perturbed. Within the perturbed cycle, there was a main effect of time (*F* (1.71, 65.14) = 8.02, *p* = 0.001, partial *η* ^2^ = 0.17, GG). Males' 50–150 ms (LLR) was lower than −50 to 0 ms (pre‐perturbed) activity in the perturbed cycle (*p* = 0.0001). Females 50–150 ms (LLR) or 150–300 ms (VR) did not differ from pre‐perturbed time bin.

In the right triceps lateral head (RTlat, Figure 8), females had a time × cycle interaction (*F* (1.24, 23.54) = 20.74, *p* < 0.0001, partial *η* ^2^ = 0.52, GG). Females' 50–150 ms (LLR) and 150–300 ms (VR) were higher in the perturbed cycle compared to non‐perturbed (*p* < 0.0001, *p* = 0.0005, respectively). Males had a time × cycle interaction (*F* (1.43, 27.12) = 8.52, *p* = 0.003, partial *η* ^2^ = 0.31, GG). Males' 50–150 ms (LLR) and 150–300 ms (VR) were higher in the perturbed cycle compared to non‐perturbed (*p* = 0.002, *p* = 0.01, respectively). Within the perturbed cycle, there was a main effect of time (*F* (1.13, 43) = 3.24, *p* < 0.001, partial *η* ^2^ = 0.37, GG). In the perturbed cycle, both females' and males' 50–150 ms (LLR) and 150–300 ms (VR) were higher than −50 to 0 ms (pre‐perturbed) activity (all *p* < 0.005). No difference between females and males during 50–150 ms (LLR) or 150–300 ms (VR) time bin in the perturbed cycle.

In the right triceps long head (RTlong, Figure 8) females had a time × cycle interaction (*F* (1.24, 23.54) = 20.74, *p* < 0.0001, partial *η* ^2^ = 0.52, GG). Females' 50–150 ms (LLR) and 150–300 ms (VR) were higher in perturbed cycle compared to non‐perturbed (*p* = 0.0002 both), and was higher than −50 to 0 ms (pre‐perturbed) activity in the perturbed cycle (both *p* < 0.005). For males, there was a main effect of time (*F* (1.07, 20.39) = 7.03, *p* = 0.01, partial *η* ^2^ = 0.27, GG). Males' 50–150 ms (LLR) was higher in perturbed cycle compared to non‐perturbed (*p* = 0.039). Males' 50–150 ms (LLR) and 150–300 ms (VR) activity were higher compared to pre‐perturbation activity in the perturbed cycle (both *p* < 0.005).

## 4 DISCUSSION

### 4.1 Tracking performance

In this study, both males and females demonstrated increased peak absolute error (AE) during perturbed cycles, confirming the efficacy of our perturbation protocol and highlighting the challenges faced by individuals when adapting to sudden changes in movement dynamics. The temporal analysis revealed nuanced differences: females initially reduced tracking error in the immediate 0–50 ms (SLR) post‐perturbation period before showing increased error at later time bins, while males demonstrated consistent increased error throughout the post‐perturbation window. These findings contribute to the ongoing debate regarding sex differences in visuomotor performance.

The mixed findings in the literature regarding sex differences in tracking accuracy likely reflect the multifactorial nature of motor performance. Some authors report a male advantage in hand tracking accuracy (Carey et al., 1994; Mathew et al., 2020; O'Brien et al., 2020), a finding that may be attributed to a variety of factors, including differences in experience and practice. For instance, regular engagement in video games can enhance visuomotor tracking abilities (Joseph & Willingham, 2000), eye‐hand coordination (Griffith et al., 1983), and reaction time (Dye et al., 2009), potentially providing males with an advantage due to reports of higher rates of gaming participation among males. However, males are more sensitive to fast‐moving stimuli with high spatial frequencies, while females exhibit greater sensitivity to slow‐moving stimuli with low spatial frequencies (Abramov et al., 2012). Given that our study focused on fast and slow tracking conditions, females may have been able to capitalize on the slower speeds to offset the higher speeds. Mathew et al. suggest that many of the observed sex differences in tracking accuracy are caused by temporal delays in reaction times for females (Mathew et al., 2020). The authors proposed that anatomical and functional sex differences in the cerebellum, which is crucial for eye‐hand coordination, may contribute to these temporal delays, as men exhibit relatively larger cerebellar regions (Raz et al., 2001) and stronger interhemispheric connectivity (Ingalhalikar et al., 2014) even after adjusting for body size. Some studies suggest that sociocultural factors, including increased participation of females in competitive sports and associated motor training, may contribute to the gradual narrowing of sex differences in visuomotor performance (Jin et al., 2023; Silverman, 2006). Importantly, some studies have reported that female athletes may experience increased androgen levels, which could enhance spatial and visuomotor abilities (Lord & Garrison, 1998) while other studies suggest that when the movement task involves the nondominant hand, females may have an advantage because they generally exhibit more symmetrical performance between their dominant and nondominant hands, whereas men show greater asymmetry and stronger right‐hand dominance in precision tasks (Liutsko et al., 2018). Notably, males demonstrated a significantly higher user rate error (movement velocity) during 50–150 ms (LLR) following the perturbation than females. This is aligned with faster decisional processing linking visual information with the movement of the arm (Dane & Erzurumluoglu, 2003; Mathew et al., 2020).

### 4.2 Neuromuscular response patterns: Feed forward Versus feedback strategies

Females consistently demonstrated higher baseline muscle activation across all triceps muscles, suggesting a proactive, co‐activation‐based approach to motor control. This strategy appears to prioritize joint stability through increased muscle stiffness, potentially providing immediate resistance to unexpected perturbations without relying on feedback‐mediated responses. In contrast, males exhibited a more reactive strategy characterized by pronounced long‐latency reflex responses, particularly in the triceps lateral head. This reactive approach may be more metabolically efficient during unperturbed movement but requires rapid neural processing and motor unit recruitment when perturbations occur. The sex‐specific activation patterns observed in different triceps heads (lateral vs. long head) may reflect biomechanical differences in joint alignment and muscle recruitment patterns creating a more effective strategy.

The biceps responses were consistent across sexes, with both groups showing inhibition post‐perturbation during the 50–150 ms (LLR) and 150–300 ms (VR) periods. This reciprocal inhibition represents an appropriate motor response to triceps stretch, suggesting that sex differences may be more prominent in agonist muscle strategies rather than in antagonist inhibition patterns. Females had higher pre‐perturbation activation of LB (~23% MVC), but an unexpected perturbation served as a high enough stimulus to inhibit motor unit activity in LB. As stated in previous research, susceptibility to inhibition is determined by the firing frequency of motor units (Miles & Türker, 1986; Uginčius et al., 2017). This inhibition is an example of context‐dependency of LLR as compared to SLR: ensuring task success in the most “efficient” way through continuous control (Crevecoeur & Kurtzer, 2018; Krutky et al., 2010). During the triceps stretch, LB was assisting in elbow flexion. Therefore, to prevent the opposing triggered elbow extension, LB as an agonist was inhibited. The mechanism is similar to the inhibition of spindle afferent group II excitatory pathways and subsequent inhibitory stretch reflex in soleus (Thompson et al., 2019) and tibialis anterior (Christensen et al., 2001) during walking.

### 4.3 Female co‐activation strategies and joint stability

Males primarily employed a reactive control strategy, characterized by larger post‐perturbation corrections, whereas females adopted a proactive, “stiff” strategy with higher baseline co‐activation. This elevated co‐activation in females is consistent with prior work demonstrating greater antagonist muscle activity during dynamic tasks (Madhavan & Shields, 2009; Osu et al., 2002; Padua et al., 2005; Saliba et al., 2020; Strazza et al., 2021), suggesting a preparatory mechanism that increases intrinsic joint stiffness and stabilizes the limb before perturbations occur (van't Veld et al., 2021). Together, these patterns indicate sex‐specific neuromuscular control strategies, with females emphasizing anticipatory stabilization and males relying more on reactive feedback to maintain movement accuracy.

### 4.4 Potential mechanisms underlying sex‐specific response patterns in antagonist muscles

Our findings indicate that males had a more consistent and pronounced triceps lateral head (LTlat) LLR (50–150 ms) to unexpected perturbation, while females did not demonstrate a prominent LLR (50–150 ms), suggesting that females may rely more on voluntary responses or co‐activation muscle strategies rather than reflexive responses in LTlat when confronted with unexpected triceps stretch. There could be several underlying factors contributing to these distinct patterns of reflexive response in triceps. For instance, males tend to have a higher proportion of fast‐twitch (type II) muscle fibers, which are associated with quick, powerful movements and are more engaged during reflexive responses like stretch reflexes (Nuzzo, 2024; Simoneau & Bouchard, 1989). Females tend to have a higher proportion of slow‐twitch (type I) fibers, which are more fatigue‐resistant and suited for endurance activities (Hunter, 2014; Wüst et al., 2008). Importantly, ovarian hormones can modulate cortical excitability, shifting control toward mechanisms involving greater cortical processing, particularly after a perturbation (Smith et al., 1999). Estrogen influences tendon laxity (Chidi‐Ogbolu & Baar, 2018; Eiling et al., 2007), proprioceptive feedback (Lee et al., 2015), and presynaptic inhibition (Hoffman et al., 2018), potentially reducing reflex pathway sensitivity and reliance on rapid, reflex‐based responses in females. Consistent with this, our previous work demonstrated menstrual cycle phase influences muscle activation and knee control (Johnson & Shields, 2024). Therefore, hormones may have contributed to the reduced LLR observed in females, leading to a greater reliance on voluntary strategies following a perturbation. Other anatomical variances between females and males may influence stretch. The median axis of the arm and forearm vary between males and females (Kholinne et al., 2018; Sharma et al., 2013) and can influence the relative muscle length during stretch.

### 4.5 Sex differences in cross‐limb responses

Sex differences in interlimb coordination suggest that females tend to have greater interhemispheric connectivity, particularly in the splenium (posterior region) of the corpus callosum (Davatzikos & Resnick, 1998). Neuroimaging studies reveal that female brains display higher interhemispheric connectivity, while male brains are structured to facilitate intra‐hemispheric cortical connectivity (Ingalhalikar et al., 2014). Greater interhemispheric connectivity in females may lead to broader, more distributed activation across motor areas. This diffuse activation pattern could reduce the amplitude or focality of EMG signals recorded from a single muscle, making localized reflex responses appear less pronounced compared to males (Fregni & Pascual‐Leone, 2006; Hodgetts & Hausmann, 2023; Spets et al., 2021; Weis et al., 2011). The implications of cross‐transcortical communication in motor learning warrant further investigation.

## 5 LIMITATIONS

Several limitations of this study should be acknowledged. The sample consisted of a relatively small group of young, healthy adults, which limits the generalizability of the findings to broader populations such as older adults or individuals with neurological or musculoskeletal impairments. While healthy participants provide a controlled framework for isolating sex‐related differences in motor control, their neuromuscular systems lack the altered excitability, compensation strategies, and impaired sensorimotor integration often observed in clinical populations. We focused our analysis on the extreme conditions (high vs. low resistance) to improve power. Future research with larger and more diverse cohorts, including those with clinical conditions, is warranted to confirm and extend these results. In addition, hormonal fluctuations and menstrual cycle phase were not controlled for, which may have influenced neuromuscular responses and contributed to variability within the female group.

## 6 CONCLUSION

This study demonstrates that males and females employ fundamentally different neuromuscular strategies when responding to unexpected perturbations during upper extremity movements. Females favor proactive co‐activation strategies that prioritize joint stability, while males utilize reactive, reflex‐based approaches that emphasize rapid error correction. The novel demonstration of sex‐specific cross‐transcortical responses provides new insights into bilateral motor control mechanisms and suggests that interhemispheric coordination may differ between sexes.

These findings advance our understanding of sex differences in motor control and highlight the importance of considering biological sex in motor control research and clinical applications. Future research should address the hormonal, biomechanical, and neural mechanisms underlying these differences while exploring their implications for training and rehabilitation strategies. Understanding these sex‐specific motor control strategies will ultimately contribute to more effective, personalized approaches to motor skill development and recovery.

## AUTHOR CONTRIBUTIONS

**Olga Dubey:** Conceptualization; data curation; formal analysis; investigation; methodology; resources; visualization. **Michael A. Petrie:** Conceptualization; data curation; formal analysis; investigation; methodology; project administration; supervision; visualization. **Richard K. Shields:** Conceptualization; data curation; formal analysis; funding acquisition; investigation; methodology; project administration; resources; supervision; validation; visualization.

## FUNDING INFORMATION

National Institute of Child Health and Human Development, Department of Physical Therapy and Rehabilitation Sciences, R01HD084645 and R01HD082109 (to RKS).

## CONFLICT OF INTEREST STATEMENT

No conflicts of interest, financial or otherwise, are declared by the authors.

## ETHICS STATEMENT

All data is deidentified and does not conflict with our human subject approval.

## Data Availability

Data will be made available upon reasonable request.
